# Representations of regular and irregular shapes by deep Convolutional Neural Networks, monkey inferotemporal neurons and human judgments

**DOI:** 10.1371/journal.pcbi.1006557

**Published:** 2018-10-26

**Authors:** Ioannis Kalfas, Kasper Vinken, Rufin Vogels

**Affiliations:** 1 Laboratorium voor Neuro- en Psychofysiologie, Department of Neurosciences, KU Leuven, Leuven, Belgium; 2 Leuven Brain Institute, Leuven, Belgium; Medical Research Council, UNITED KINGDOM

## Abstract

Recent studies suggest that deep Convolutional Neural Network (CNN) models show higher representational similarity, compared to any other existing object recognition models, with macaque inferior temporal (IT) cortical responses, human ventral stream fMRI activations and human object recognition. These studies employed natural images of objects. A long research tradition employed abstract shapes to probe the selectivity of IT neurons. If CNN models provide a realistic model of IT responses, then they should capture the IT selectivity for such shapes. Here, we compare the activations of CNN units to a stimulus set of 2D regular and irregular shapes with the response selectivity of macaque IT neurons and with human similarity judgements. The shape set consisted of regular shapes that differed in nonaccidental properties, and irregular, asymmetrical shapes with curved or straight boundaries. We found that deep CNNs (Alexnet, VGG-16 and VGG-19) that were trained to classify natural images show response modulations to these shapes that were similar to those of IT neurons. Untrained CNNs with the same architecture than trained CNNs, but with random weights, demonstrated a poorer similarity than CNNs trained in classification. The difference between the trained and untrained CNNs emerged at the deep convolutional layers, where the similarity between the shape-related response modulations of IT neurons and the trained CNNs was high. Unlike IT neurons, human similarity judgements of the same shapes correlated best with the last layers of the trained CNNs. In particular, these deepest layers showed an enhanced sensitivity for straight versus curved irregular shapes, similar to that shown in human shape judgments. In conclusion, the representations of abstract shape similarity are highly comparable between macaque IT neurons and deep convolutional layers of CNNs that were trained to classify natural images, while human shape similarity judgments correlate better with the deepest layers.

## Author summary

The primate inferior temporal (IT) cortex is considered to be the final stage of visual processing that allows for object recognition, identification and categorization of objects. Electrophysiology studies suggest that an object’s shape is a strong determinant of the neuronal response patterns in IT. Here we examine whether deep Convolutional Neural Networks (CNNs), that were trained to classify natural images of objects, show response modulations for abstract shapes similar to those of macaque IT neurons. For trained and untrained versions of three state-of-the-art CNNs, we assessed the response modulations for a set of 2D shapes at each of their stages and compared these to those of a population of macaque IT neurons and human shape similarity judgements. We show that an IT-like representation of similarity amongst 2D abstract shapes develops in the deep convolutional CNN layers when these are trained to classify natural images. Our results reveal a high correspondence between the representation of shape similarity of deep trained CNN stages and macaque IT neurons and an analogous correspondence of the last trained CNN stages with shape similarity as judged by humans.

## Introduction

Recently, several studies compared the representations of visual images in deep Convolutional Neural Networks (CNN) with those of biological systems, such as the primate ventral visual stream [1–4]. These studies showed that the representation of visual objects in macaque inferior temporal (IT) cortex corresponds better with the representations of these images in deep CNN layers than with representations of older computational models such as HMAX [5]. Similar findings were obtained with human fMRI data [6–10]. The images used in these studies were those of real objects in cluttered scenes, which are the same class of images as those employed to train the deep CNNs for classification. Other single unit studies of IT neurons employed two-dimensional (2D) shapes and observed highly selective responses to such stimuli (for review see [11]). If deep CNNs provide a realistic model of IT responses, then the CNNs should capture also the selectivity observed for such two-dimensional shapes in IT. To our knowledge, thus far there has been no comparison between the 2D-shape representation of IT neurons, measured with such reduced stimuli, and that of deep CNN models. It is impossible to predict from existing studies that compared deep CNN activations and neurophysiology whether the deep CNNs, which are trained with natural images, can faithfully model the selectivity of IT neurons for two-dimensional abstract shapes. Nonetheless, such correspondence between CNN models and single unit selectivity for abstract shapes is critical for assessing the generalizability of CNN models to stimuli that differ markedly from those of the trained task but have been shown to drive selectively IT neurons.

Previously, we showed that a linear combination of units of deep convolutional layers of CNNs trained with natural images could predict reasonably well the shape selectivity of single neurons recorded from an fMRI-defined body patch [4]. However, in that study, we adapted for each single unit the shapes to the shape preference of that neuron, precluding a comparison between the shape representation of the population of IT neurons and deep CNNs. To perform such a comparison, one should measure the responses of IT neurons to the same set of shapes. Furthermore, the shape set should include variations in shape properties IT neurons were shown to be sensitive to. Also, the IT response selectivities for such shapes should not trivially be explainable by physical image similarities, such as pixel-based differences in graylevels.

**Fig 1:**
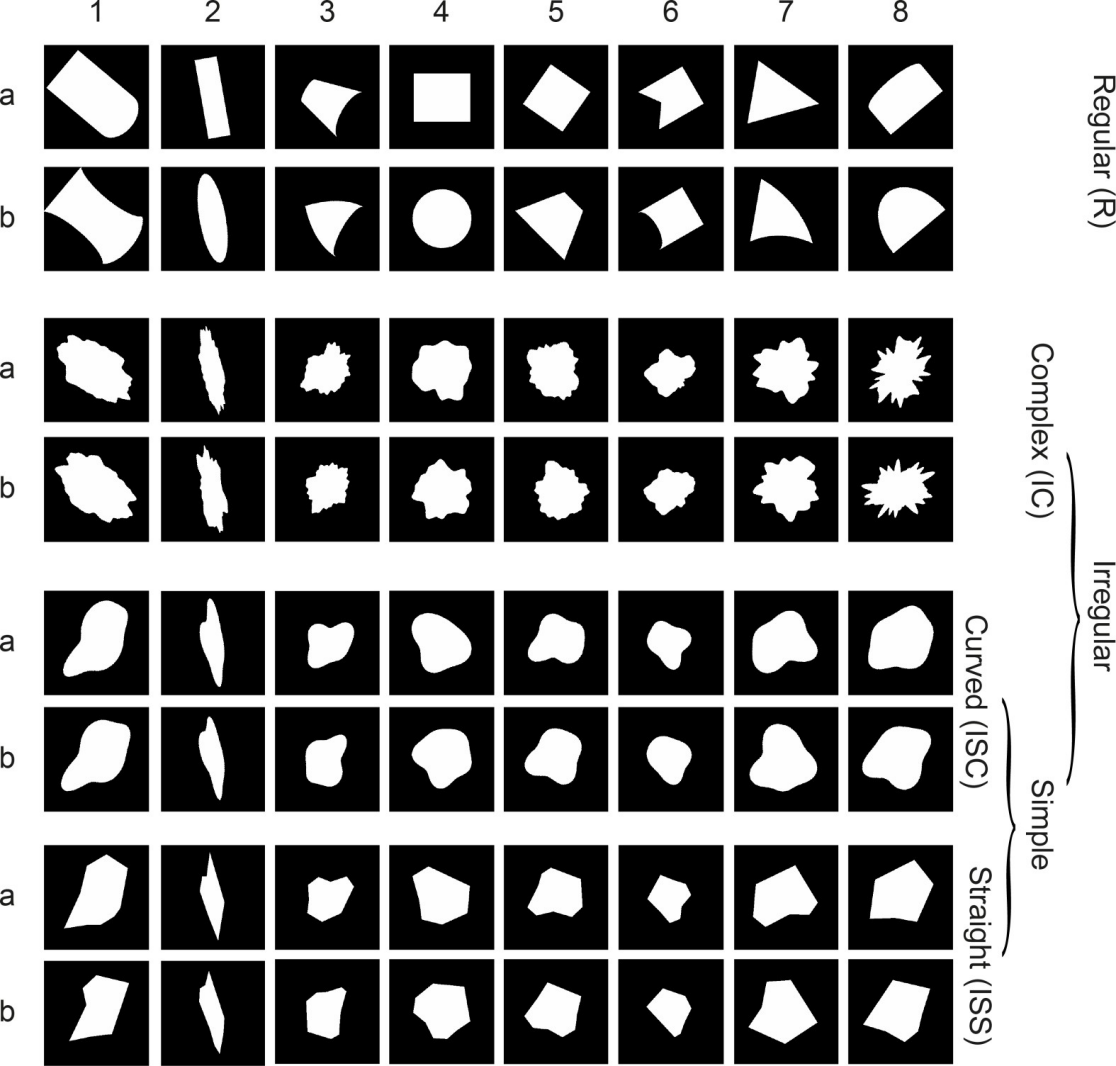
Shape set. One group of Regular (R) and three groups of Irregular shapes: Irregular Complex (IC), Irregular Simple Curved (ISC) and Irregular Simple Straight (ISS). A group of 16 shapes corresponds to two consecutive rows (labeled a,b) and the group names are depicted in the tree graph on the right. The 8 pairs (a,b) of each group are defined by the numbers on top of the figure (1,2, …8).

Kayaert et al. [12] measured the responses of single IT neurons to a set of shapes that varied in regularity and the presence of curved versus straight boundaries (Fig 1). The first group of stimuli of [12] was composed of regular geometric shapes (shown in the first two rows of Fig 1 and denoted as Regular (R)) that all have at least one axis of symmetry. These shapes are simple, i.e., have low medial axis complexity [13]. The stimulus pairs in each column of these two rows (denoted by *a* and *b*) differed in a non-accidental property (NAP). NAPs are stimulus properties that are relatively invariant with orientation in depth, such as whether a contour is straight or curved or whether a pair of edges is parallel or not. These properties can allow efficient object recognition at different orientations in depth not previously experienced [14–16]. NAPs can be contrasted with metric properties (MPs), which vary with orientation in depth, such as aspect ratio or the degree of curvature. The three other groups are all ‘Irregular’. They differed from the Regular shapes in that they do not have a single axis of symmetry. The two shapes in each row of the three Irregular groups differed in the configuration of their concavities and convexities or corners. The shapes in the Irregular Simple Curved (ISC) set all had curved contours. The Irregular Simple Straight (ISS) shapes were derived from the ISC shapes by replacing the curved contours with straight lines. Thus, the corresponding stimuli in the ISS and ISC shapes differed in a NAP. Last, the Irregular Complex (IC) group was more complex in that the shapes in that group had a greater number of contours.

Kayaert et al. [12] found that anterior IT neurons distinguished the four groups of shapes. Importantly, the differences in IT responses amongst the shapes could not be explained by pixel-based gray level differences, nor by HMAX C2 unit differences. In fact, none of the tested quantitative models of object processing could explain the IT response modulations. Furthermore, the IT response modulations were greater for the Regular shapes and when comparing the curved and straight Irregular Simple shapes than within the 3 Irregular shape groups, suggesting a greater sensitivity for NAPs than for MPs (see also [17,18]). We reasoned that this shape set and corresponding IT responses was useful to examine to what degree different layers of deep CNNs and IT neurons represent abstract shapes similarly. We employed deep CNNs that were pretrained to classify ImageNet data [19], consisting of images of natural objects in scenes. Hence, the CNNs were not exposed during training to silhouette shapes shown to the IT neurons. Deep CNNs have a particular architecture with early units having small receptive fields, nonlinear pooling of units of the previous layer, etc. Such a serial, hierarchical network architecture with increasing receptive field size across layers may result in itself, i.e. without training, in changes in the representational similarity across layers. To assess whether potential correlations between IT and CNN layer response modulations resulted from classification training or from the CNN architecture per se, we also compared the activations of untrained CNNs with the IT response modulations.

Kayaert et al. [12] had also human subjects sort the same shapes based on similarity and found that human subjects had a pronounced higher sensitivity to the difference between the curved and straight simple irregular shapes (relative to the regular shapes) than the IT neurons. We examined whether a similar difference in response pattern between macaque IT neurons and human similarity judgements would emerge in the deep CNNs. We expected that deeper layers would resemble the human response patterns while the IT response pattern would peak at less deep layers.

## Results

**Fig 2:**
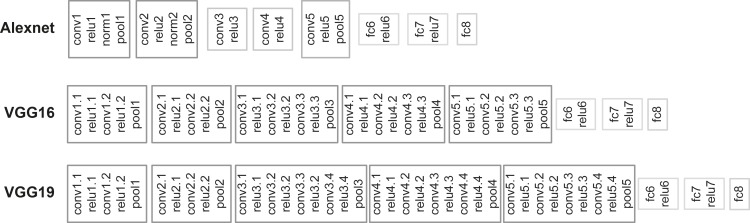
Model architectures. Deep Convolutional Neural Network (CNN) architectures for the 3 different networks that we employed: Alexnet, VGG-16 and VGG-19. Early computational blocks consist of consecutive operations such as: convolution (conv), RELU activation function, normalization (norm; only for Alexnet) and max pooling (pool). The later stages of each CNN incorporate three fully connected (fc) layers, where the first two are followed by a RELU activation function.

Kayaert et al [12] recorded the responses of 119 IT neurons to the 64 shapes shown in Fig 1. The 64 shapes are divided in four groups based on their regularity, complexity and whether they differed in NAPs. We presented the same shapes to 3 deep CNNs: Alexnet [20], VGG-16, VGG-19 [21] and measured the activations of the units in each layer of the deep nets. These deep nets differ in their number of layers, the number of units in each layer and the presence of a normalization stage, but each have rectifying non-linearity (RELU) and max pooling stages (Fig 2). We employed deep nets that were pre-trained in classification of a database of natural images, which were very different in nature from the abstract shape stimuli that we employ here to test the models and neurons. The aim was to compare the representations of the shapes between IT neurons and each layer of the deep nets. To do this, we employed representational similarity analyses [22,23], following the logic of second order isomorphism [24,25], and examined the correlation between the neural IT-based similarities and CNN-based similarities in responses to shapes. We are not trying to reconstruct the shapes based on IT neuron or CNN unit outputs but we are examining whether shapes that are represented close to each other in the neural IT space are also represented close to each other in the CNN layer space.

**Fig 3:**
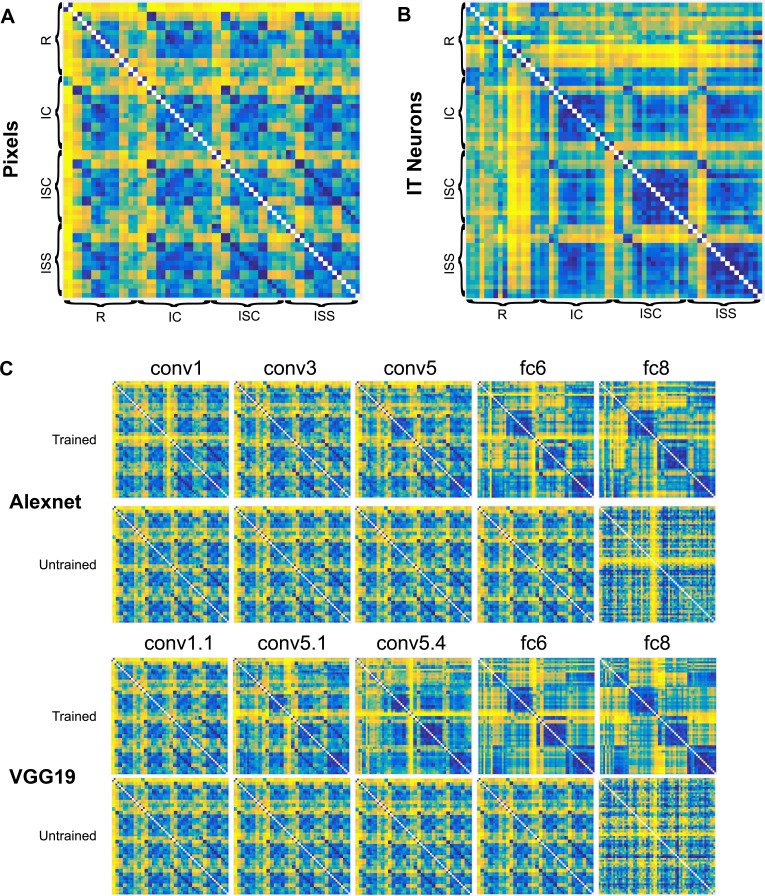
Dissimilarity matrices. Matrices of Euclidean distances for pixel gray-levels (A), the IT neurons (B), and 5 layers of the trained and untrained versions of 2 deep CNNs (C). Note that the dissimilarity matrices are by definition symmetric about the diagonal of zeros, which is plotted in white color. The stimulus groups are indicated in (A) as in Fig 1 and the CNN layers in (C) have the same terminology as in Fig 2. The matrices have been separately normalized and are plotted in percentile units, following [23]. Dissimilarities increase from blue to yellow.

In a first analysis, we computed the pairwise dissimilarity between all 64 stimuli using the responses of the IT neurons and the activations in each of the CNN layers. We employed two dissimilarity metrics: Euclidean distance and 1 –Spearman rank correlation ρ. The dissimilarity matrices computed with the Euclidean distance metric for the IT neurons and for 5 layers of the trained CNNs are illustrated in Fig 3B and 3C, respectively. In this and the next figures, we will show only the data for Alexnet and VGG-19, since VGG-16 and VGG19 produced similar results. In addition, Fig 3A shows the pixel-based dissimilarities for all image pairs. Visual inspection of the dissimilarity matrices suggests that (1) the pattern of dissimilarities changes from the superficial to deep layers in a relatively similar way in the CNNs, (2) the dissimilarity matrix of the first layer (e.g. conv1.1) resembles the pixel-based similarities (Fig 3A) and (3) the deeper layers resemble more the IT neural data (Fig 3B).

**Fig 4:**
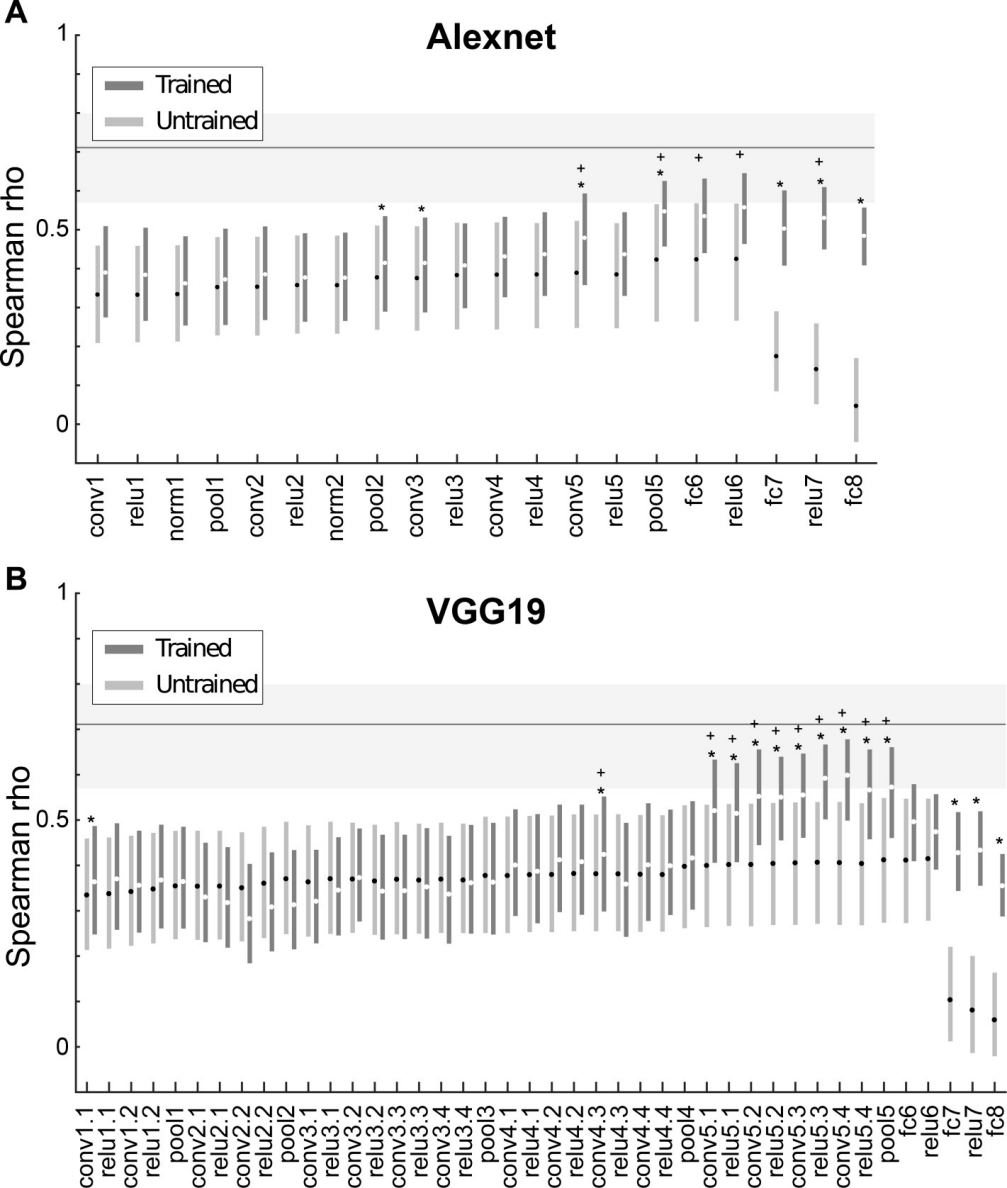
Representational similarity analysis of deep CNN layers and IT neurons for the whole shape set. Spearman rank correlation coefficients between IT and model layer similarities are shown for each layer of Alexnet (A) and VGG19 (B) using the Euclidean distance metric. Error bars depict 95% confidence intervals, determined by 10,000 bootstrap samples of the IT neuron pool (n = 119 neurons). Stars indicate layers for which the Spearman rank correlations for the trained version differed significantly from its untrained version (paired bootstrap test (see Materials and Methods); False Discovery Rate corrected q<0.05). Crosses indicate trained layers which differed significantly from the first convolutional layer of the network (paired bootstrap test (see Materials and Methods); False Discovery Rate corrected q<0.05). Layers are indicated by the same nomenclature as in Fig 2. The horizontal line and gray band indicate the median and 95% interval, respectively, of the Spearman-Brown corrected split-half correlations (n = 10000 splits) of the neuronal distances, as an estimate of the noise ceiling.

We quantified the similarity between the IT shape representation and that of each layer by computing the Spearman Rank correlation between the corresponding pairwise dissimilarities of IT and each layer. Thus, we could assess to what degree stimuli that produce a very different (similar) pattern of responses in IT also show a different (similar) pattern of activations in a CNN layer. We found that for both dissimilarity metrics the similarity between IT neuronal responses and trained CNN layer activations increased significantly with the depth of the layer. This is shown using the Euclidean distance metric for Alexnet and VGG-19 in Fig 4 (see S1 Fig for the data of both distance metrics and the 3 networks). In the VGG nets, the similarity peaked at the deepest convolutional layers (Fig 4) and then decreased for the deepest layers. In fact, the Spearman correlations for the last two fully connected layers did not differ significantly from that of the first convolutional layer in each CNN (Fig 4). The decrease in similarity for the deepest layers was weaker in Alexnet. The peak similarity was similar amongst the 3 nets, with ρ hovering around 0.60, and were larger for the correlation (mean peak ρ = 0.64) compared with Euclidean distance metric (mean peak ρ = 0.58). To assess the degree to which the models explained the neural data, we computed the reliability of the neural-based distances giving the finite sampling of the IT neuron population. This noise ceiling was computed by randomly splitting the neurons into two groups, computing the dissimilarities for each group, followed by computation of the Spearman rank correlation between the dissimilarities of the two groups. This split-half reliability computation was performed for 10000 random splits. Fig 4 shows the 2.5, 50 (median) and 97.5 percentiles of the Spearman-Brown corrected correlations between the two groups. The correlations between (some) CNN layers and neural responses were close but still below the estimated noise of the neural dissimilarities.

In order to assess to what degree the similarity between neural data and the CNN layers reflects the architecture of the CNNs versus image classification training, we computed also the similarity for untrained networks with random weights. Fig 3C illustrates dissimilarity matrices computed using Euclidean distances for 5 untrained layers of two CNNs. Visual inspection suggests little change in the dissimilarity matrices of the different layers of the CNNs, except for fc8. Furthermore, the pattern of dissimilarities resembled the pixel-based dissimilarities shown in Fig 3A. Both observations were confirmed by the quantitative analysis. The Spearman correlations of the neural data and untrained CNNs increased only weakly with depth, except for a marked decrease in correlation for the last two fully connected layers. Except for the deep convolutional and the last two layers, the trained and untrained networks showed similar Spearman correlations of the neural and CNN distances (Fig 4). This suggests that overall the similarity between the IT data and the shallow CNN layers are unrelated to classification training but reflect merely the CNN architecture. Significant differences between trained and untrained CNNs were observed for the deeper convolutional layers (Fig 4), suggesting that the similarity between IT and the deep convolutional layers depends on classification training. The similarities for the first fully connected layer (fc6 and relu6 in Fig 4) did not differ significantly between the trained and untrained layers (except for the correlation metric in AlexNet (S1 Fig). The deepest two (fully connected) layers showed again a significantly greater similarity for the trained compared with the untrained networks. However, this can be the result of the sharp drop in correlations for these layers in the untrained network. Overall, these data suggest that the shape representations of the trained deep convolutional layers, but not of the deepest layers, shows the highest similarity with shape representations in macaque IT.

**Fig 5:**
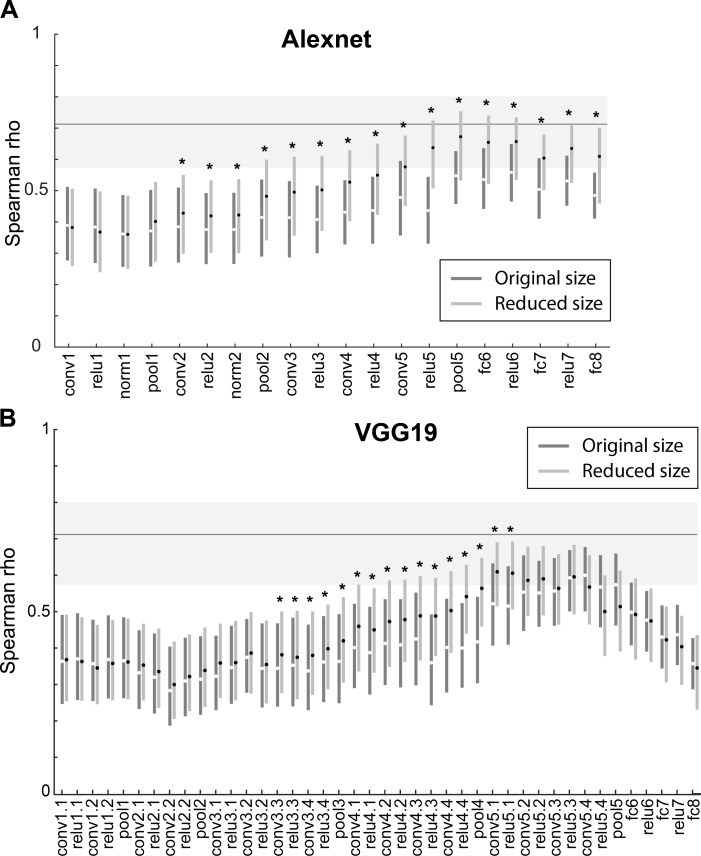
Representational similarity analysis of deep CNN layers and IT neurons for the whole shape set with two different sizes. Spearman rank correlation coefficients between IT and model layer similarities are shown for each layer of Alexnet (A) and VGG19 (B) for the original and twofold smaller sizes (“reduced size”). The dissimilarities were Euclidean distances. Error bars depict 95% confidence intervals, determined by 10,000 bootstrap samples of the IT neuron pool (n = 119 neurons). Stars indicate layers for which the Spearman rank correlations for the trained version differed significantly from its untrained version (paired bootstrap test; False Discovery Rate corrected q<0.05). The horizontal line and gray band indicate the median and 95% interval, respectively, of the Spearman-Brown corrected split-half correlations (n = 10000 splits) of the neuronal distances, as an estimate of the noise ceiling.

Receptive field (RF) size increases along the layers of the CNNs, allowing deeper layer units to integrate information from larger spatial regions. The difference in IT-CNN similarity between untrained and trained layers shows that the increase in RF size cannot by itself explain the increased IT-CNN similarity in deeper layers, since untrained CNN also increase their RFs along the layer hierarchy. Also, the decrease in similarity between IT responses and the fully connected layers argues against RF size being the mere factor. Nonetheless, although not the only contributing factor, RF size is expected to matter since arguably small RFs cannot capture overall shape when the shape is relatively large. Hence, it is possible that the degree of IT-CNN similarity for different layers depends on shape size, with smaller shapes showing a greater IT-CNN similarity at earlier layers. We tested this by computing the activations to shapes that were reduced in size by a factor of two in all layers of each of the 3 trained CNNs. Fig 5 compares the correlations between dissimilarities of the trained Alexnet and VGG-19 networks and IT dissimilarities for the original and reduced sizes, with dissimilarities computed using Euclidean distances. The stars indicate significant differences between the similarities for the two sizes (tested with a FDR corrected randomization test; same procedure as in Fig 4 when comparing trained and untrained correlations). In each of the CNNs (S2 Fig), the IT-CNN similarity increased at more superficial layers for the smaller shape. The overall peak IT-CNN similarity was highly similar for the two sizes in the VGG networks and occurred at the deep convolutional layers. For Alexnet, the overall similarity was significantly higher for the smaller shapes in the deep layers. This analysis indicates that shape size is a contributing factor that determines at which layer the IT-CNN similarity increases, but that for the VGG networks, peak similarity in the deep layers does not depend on size (at least not for the twofold variation in size employed here). Note that also for the smaller size the IT-CNN similarity drops markedly for the fully connected layers in the VGG networks. Thus, the overall trends are independent of a twofold change in shape size.

**Fig 6:**
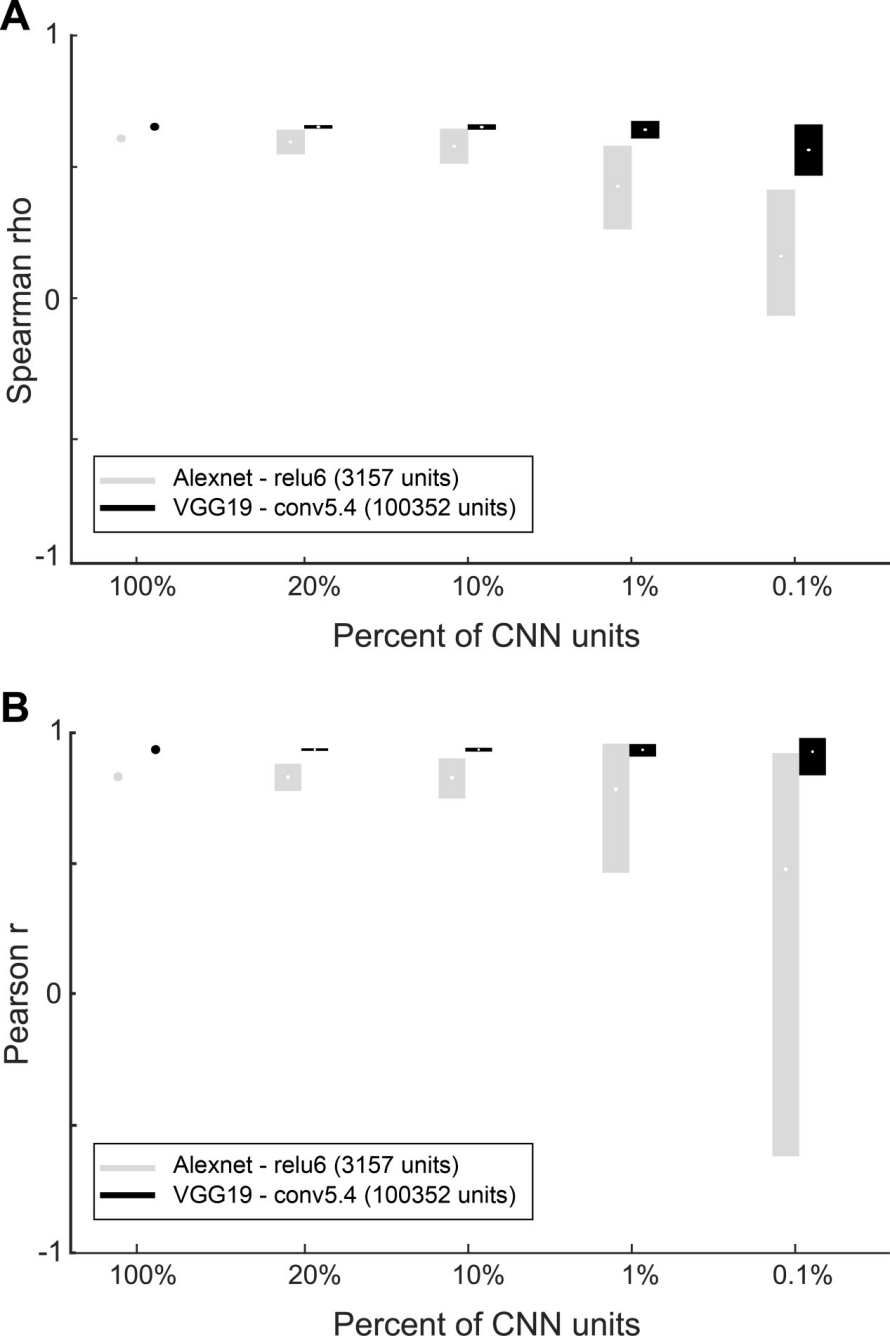
Similarities between IT and CNN peak layer shape dissimilarities as a function of percent of units. (A) Spearman rank correlation coefficients between IT and peak CNN layer similarities are shown for each of two CNN models as a function of sample size, expressed as percentage of the total number of units that were activated differentially by the 64 shapes. (B) Pearson correlation coefficients between the mean neural distances and the mean distances of the peak CNN layer (n = 6 mean distances; Fig 10) as a function of percentage of the total number of units. The total number of units (100%) for each CNN layer is listed in the legend. Note that 0.1% corresponds to only 3 Alexnet units, explaining the large range of correlations for that sample size. The dissimilarities were Euclidean distances. Error bars depict 95% confidence intervals, determined by 10,000 random samples from the population of differentially activated CNN units of that layer.

In the preceding analyses, we included all units of each CNN layer. To examine whether the similarity between the CNN layers and the IT responses depends on a relatively small number of CNN units or is distributed amongst many units, we reran the representational similarity analysis of deep CNN layers and IT neurons for the whole shape set for smaller samples of CNN units. We took for each network the layer showing the peak IT-CNN similarity and for that layer sampled 10000 times at random a fixed percentage of units. We restricted the population of units to those that showed a differential activation (standard deviation of activation across stimuli greater than 0) since only those can contribute to the Euclidean distance. Fig 6A plots the median and 95% range of Spearman rank correlation coefficients between IT and CNN layer dissimilarities for the whole shape set as a function of the percent of sampled units for two CNNs. We found that the IT-CNN similarity was quite robust to the number of sampled units. For instance, for Alexnet, the IT-CNN similarity for the original and the 95% range of the 10% samples overlap, indicating that 315 Alexnet units can produce the same IT-CNN similarity as the full population of units. Note also that the lower bound of the 95% range is still above the IT-CNN similarities observed for the untrained network (median Spearman rho about 0.40; see Fig 4). This indicates that the IT-CNN similarity does not depend on a small subset of units, since otherwise the range of similarities (Spearman rho correlations) for the 10% samples would be much greater. The same holds for the other CNNs (S3 Fig), except that these tolerated even smaller percent sample size (for VGG19 even 0.1%, which corresponds to 100 units).

**Fig 7:**
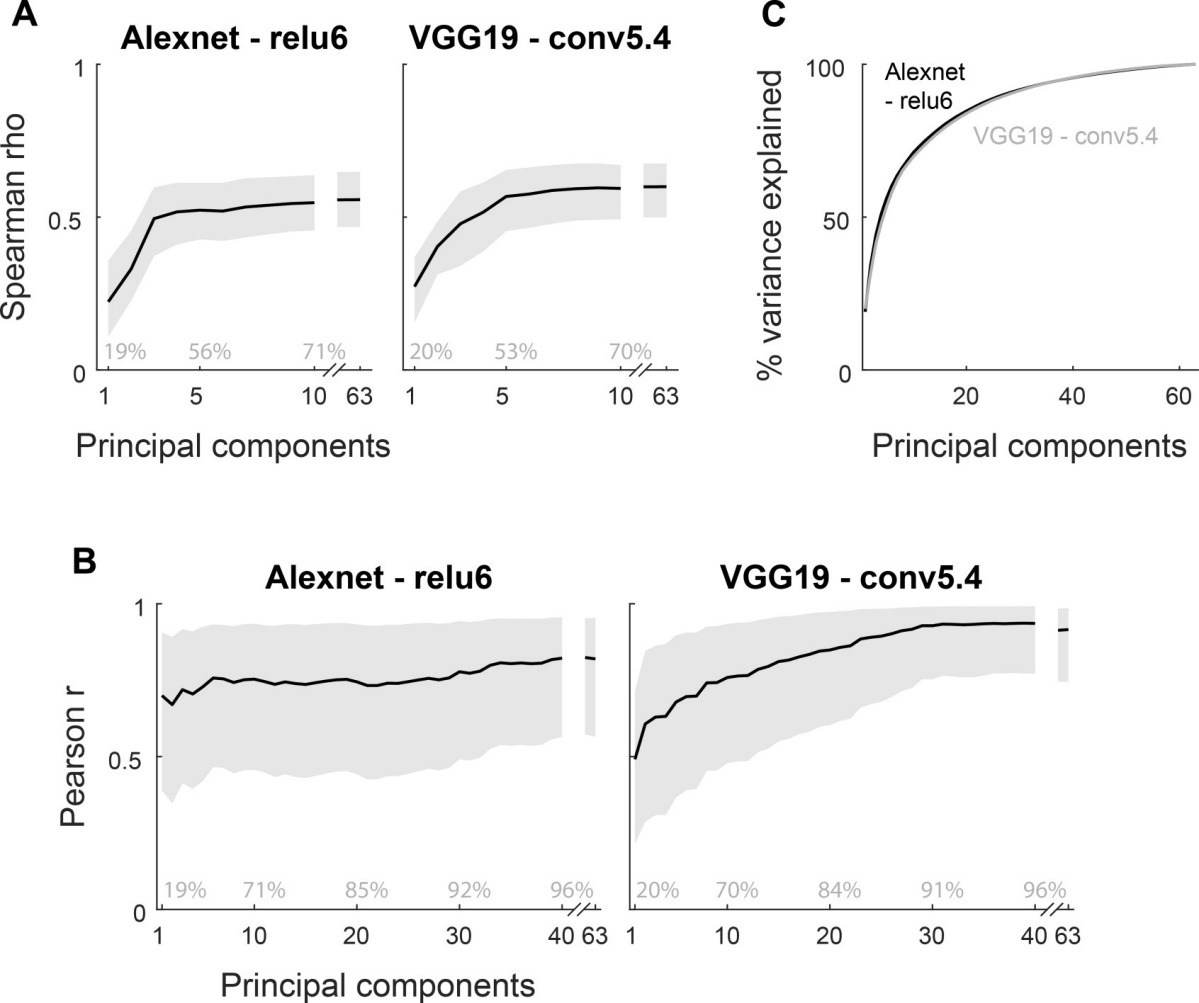
Similarities between IT and CNN peak layer shape dissimilarities as a function of retained principal components. (A) Spearman rank correlation coefficients between IT and peak CNN layer similarities are shown for each of two CNN models as a function of retained principal components of the CNN layer activations. The dissimilarities were Euclidean distances. Error bands depict 95% confidence intervals, determined by 10,000 bootstrap samples of the IT neuronal pool. (B) The cumulative proportion of explained variance as a function of principal component number for the Alexnet (black line) and VGG-19 layer (gray line). (C) Pearson correlation coefficients between the mean neural distances and the mean distances of the peak CNN layer (n = 6 mean distances; see Fig 10) as a function of retained principal components. In A and C, the bands represent 95% confidence intervals, determined by 10,000 bootstrap samples of the IT neuronal pool.

The above analysis appears to suggest that the activations of the CNN units to the shapes are highly correlated with each other. To address this directly, we performed Principal Component Analysis (PCA) of unit activations of the same peak CNN layers as in Fig 6 and computed Euclidean distance based dissimilarities between all stimulus pairs for the first, first two, etc. principal components (PCs), followed by correlation with the neural dissimilarities as done before for the distances computed across all units of a CNN layer. For both the Alexnet and VGG-19 layer, the first 10 PCs explained about 70% of the variance in CNN unit activations to the 64 stimuli (Fig 7B). Only the first 3 (Alexnet) or 5 (VGG-19) PCs were required to obtain a similar correlation between the model and neural distances as observed when using all model units of the layer (Fig 7A; about 7 PCs were required for VGG-16; see S4 Fig). This analysis shows that the neural distances between the abstract shapes relate to a relatively low dimensional shape representation in the CNN layer, with a high redundancy between the CNN units.

**Fig 8:**
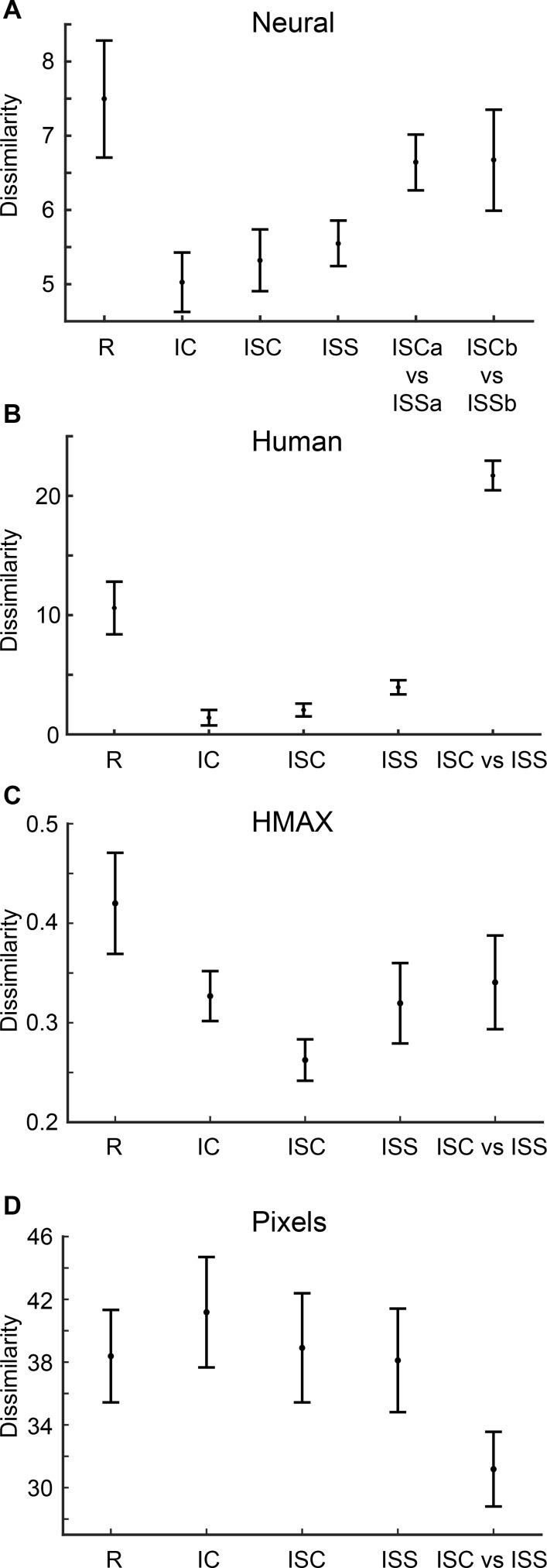
Response modulations for the shape groups: IT neurons, human judgements, HMAX and pixel-based dissimilarities. (A) Mean response modulations of IT neurons for the shape groups R, IC, ISC, ISS, “ISCa vs ISSa” and “ISCb vs ISSb”. See Fig 1 for the nomenclature of the different shape groups. (B) Dissimilarities for the shape groups (R, IC, ISC, ISS, ISC vs ISS) based on human judgements. (C) Dissimilarities for the same shape groups based on the HMAX C2 layers’ output. (D) Pixel-based dissimilarities. Error bars indicate standard errors of the mean. B, C and D are taken from Kayaert et al. [12].

In the above analyses, we compared the overall similarity of the shape representations in IT and CNN layers. However, a more stringent comparison between the shape representations in IT and the CNNs involves response modulations for the shape pairs for which Kayaert et al [12] observed striking differences between predictions of pixel-based models or computational models like HMAX and the neural responses. The average response modulations (quantified by pairwise Euclidean distances) for the different group pairs comparisons are shown in Fig 8 for the IT neural data, the HMAX C2 layer and the pixel differences. Kayaert et al [12] showed that the mean response modulation in IT (Fig 8A)was significantly greater for the regular shape pairs (1–8 in Fig 1) than for the 3 irregular shape group pairs, despite the pixel differences between members of a pair being, on average, lower or similar for the regular group than for the 3 irregular groups (Fig 8D). In addition, the response modulation to ISC vs. ISS was significantly greater than the modulations within IC, ISC and ISS, although the average pixel-difference within the ISC vs. ISS-pairs was much lower than the pixel-differences within the other pairs. This differential neural response modulation to ISC vs ISS was present for both members of the ISC and ISS pairs (a and b members: “ISCa vs ISSa” and “ISCb vs ISSb”) and thus was highly reliable. Note that the difference between ISC vs. ISS and the IC and ISS shape groups that are present in the neural data is not present for the HMAX C2 distances (Fig 8C). Kayaert et al. [12] reported also a relatively higher sensitivity to the straight vs. curved contrast of the ISC vs. ISS comparison compared with the regular shapes in human similarity ratings (Fig 8B), compared with the IT neural data. In other words, human subjects appear to be more sensitive to the curved versus straight NAP difference than macaque IT neurons.

**Fig 9:**
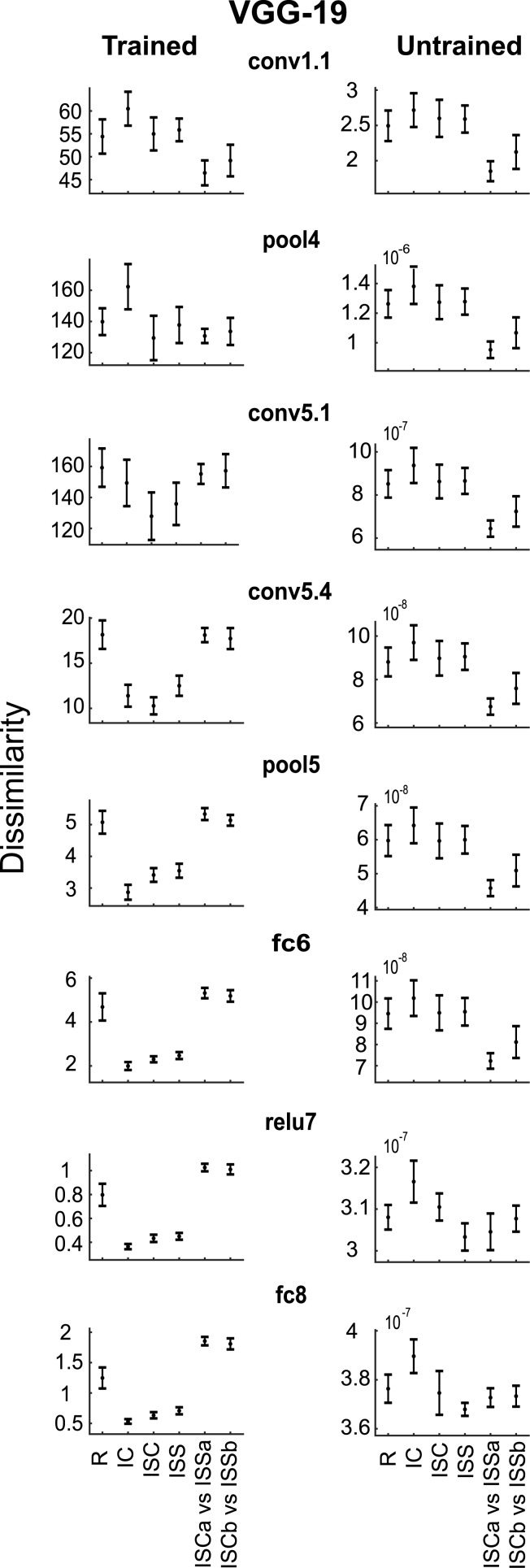
Response dissimilarities for the shape groups: Deep CNN layers. Dissimilarities for groups R, IC, ISC, ISS, “ISCa vs ISSa”, “ISCb vs ISSb” of selected trained (left column) and untrained (right column) versions of VGG-19 layers (same nomenclature as in Fig 2). Same conventions as in Fig 8. The specific selection of layers is motivated by the fact that there were no critical differences in the layers that follow the selected ones.

In a second analysis, we determined whether the marked differences in IT response modulations and human judgements shown in Fig 8 are present in the dissimilarities for the different layers of the deep CNNs. Fig 9 illustrates the results for 8 layers of VGG-19. The left column of the figure plots the distances for the trained network. The dissimilarities for the first convolutional layer fits the pixel-based distances amongst the shape pairs (Fig 8D; Pearson correlation between pixel-based distances and first layer distances = 0.966), but differ from those observed in IT and for human judgements. Similar trends are present until the very deep convolutional layers where the dissimilarities became strikingly similar to those observed in macaque IT (e.g. compare trained conv5.4 or pool5 of Fig 9 with Fig 8A). The dissimilarities for the last two layers (e.g. trained relu7 and fc8 in Fig 9) are strikingly similar to those observed for the human judgements (Fig 8B), and differ from the pattern seen in macaque IT neurons. Indeed, as noted above, the human judgements differ from the IT responses in their sensitivity for the ISC vs ISS comparison relative to that for the regular shape pairs: for the human judgement distances, the ISC vs ISS distances are greater than for the regular shape distances while for the neural distances both are statistically indistinguishable (Kayaert et al. [12]). Therefore, we tested statistically for which CNN layer the ISC vs ISS distances were significantly greater than the regular shape distances (Wilcoxon test), thus mimicking the human distances. We found a significant difference for the very deep VGG19 layer fc8 (p = 0.039) and VGG16 layers fc7 (p = 0.039), relu7 (p = 0.023), and fc8 (p = 0.023). Although the deepest Alexnet (fully connected) layers showed the same trend, this failed to reach significance. These tests showed that only the very deep CNN layers mimicked the human judgements. None of the untrained CNN layers showed a dissimilarity profile similar to that observed in monkey IT or in human judgements (Fig 9, right column). In fact, the untrained data resembled more the pixel-based distances (see Fig 8D). Indeed, the Pearson correlation between the pixel-based distances and the conv1.1 distances was 0.999 for the untrained VGG-19.

**Fig 10:**
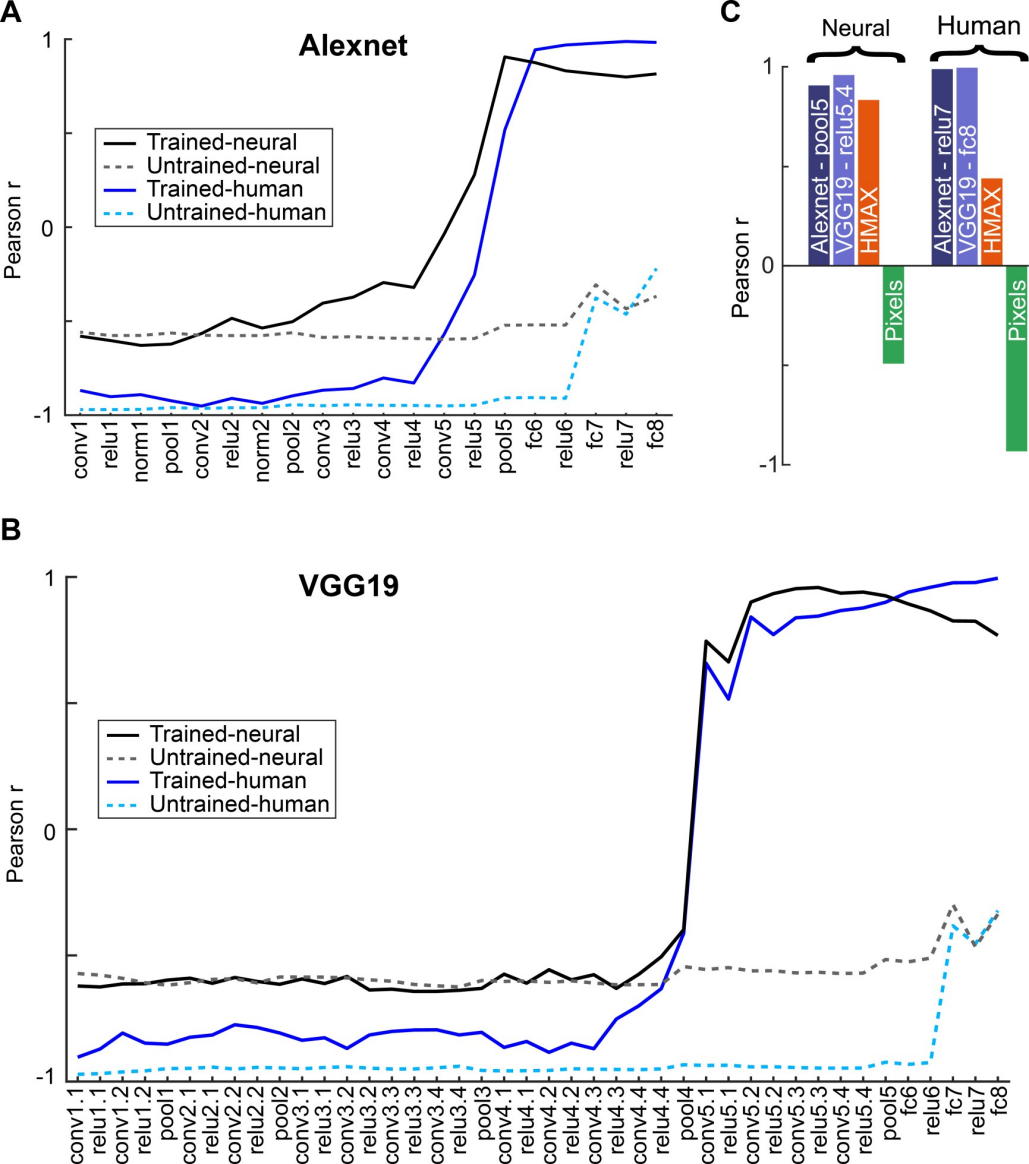
Correspondence between model dissimilarities and biological dissimilarities (IT responses and human judgement-based dissimilarities) for the shape groups. (A, B). Gray curves show the Pearson correlation coefficients between the mean neural distances and the mean distances of the CNN layers (n = 6 mean distances per layer). Blue curves show the Pearson correlation coefficients of the CNN layer distances and the distances based on human judgements. Data for trained and untrained CNNs are plotted with full and dashed lines, respectively. Nomenclature of CNN layers as in Fig 2. Results for all two models (Alexnet and VGG-19) are displayed in the subplots (A, B). (C) Neural: Pearson correlation coefficient between the mean IT distances and the mean distances of the peak Alexnet layer, peak VGG-19 layer, the mean HMAX C2 layer distances, and mean pixel-based distances, across shape groups. Human: Pearson correlation coefficient between the distances based on the human judgements and the peak Alexnet layer, peak VGG-19 layer, HMAX C2 layer and pixel-based distances.

We quantified the correspondence between the neural response dissimilarities of Fig 8A and the CNN layer dissimilarities (as in Fig 9) by computing the Pearson correlation coefficient between the dissimilarity profiles (n = 6 dissimilarity pairs). The same quantification was performed for the human judgements (Fig 8B) and the CNN dissimilarities (n = 5 pairs). These correlations are plotted in Fig 10A and 10B as a function of layer for two CNNs, trained and untrained. For the neural data, the correlations are negative for the shallow layers and highly similar for the trained and untrained CNNs. The negative correlations are a result of the nearly inverse relationship between neural and low-level (pixel) differences between the shapes (Fig 8D). This was not accidental, but by design: when creating the stimuli, Kayaert et al [12] ensured that the NAP differences (e.g. between ISC and ISS) were smaller than MP differences. For both VGG networks (S5 Fig; Fig 10B), there was a sharp increase in correlations at the trained deep 5.1 convolutional layer, followed by a decrease of the correlations for the fully connected layers. This trend was similar, although more abrupt, to that observed for the global dissimilarities of Fig 4. For Alexnet, the increase of the correlations with increasing depth of the trained convolutional layers was more gradual, but like the VGG networks, high correlations were observed for the deeper trained convolutional layers. For the human judgement data, the correlations were already higher for the trained compared with the untrained CNNs at the shallow layers, although still negative. Like the neural data, there was a marked increase in correlation at the very deep trained layers. Contrary to the neural data, the correlations for the human judgements continued to increase along the trained fully connected layers, approaching a correlation of 1 at the last layer. These data show that the average response modulations of IT neurons for the shape groups of Fig 1 correspond nearly perfectly with those of the deeper layers of CNNs, while the differences in human similarity judgements between the groups are captured by the later fully connected layers of the CNNs. This holds for Alexnet and VGG nets. Note that the deep CNN layers performed better at predicting the neural IT and human perceptual dissimilarities than the HMAX C2 layer output (Fig 10C).

As for the representational similarity analysis for all shapes (Fig 6A), we computed also the Pearson correlation coefficients between the dissimilarity profiles (n = 6 dissimilarity pairs) of the same peak CNN layers and the IT distances for the 6 shape groups (as in Fig 10) for smaller samples of units. As shown in Fig 6B, we observed similar IT-CNN correlations for the within-group distances up to the 1% and 0.1% samples compared with the full population of units for Alexnet and VGG, respectively. Again, this suggests that IT-CNN similarity does not depend on a small number of outlier CNN units. The greater tolerance for percent sample size for the VGG units is because the VGG layers consisted of a larger number of units per se (total number of units are indicated in the legend of Fig 6). In addition, we computed the mean distances for the same layers and their correlation with the mean neural modulations as a function of retained PCs (Fig 7B). Up to 30 PCs were required to obtain a similar correlation between neural and CNN layer distances for the six groups of shapes as when including all units of the layer (Fig 7B). This suggests that the close to perfect modeling of the mean response modulations across the 6 shape groups required a relatively high dimensional representation of the shapes within the CNN layer.

## Discussion

The particular set of shapes that we employed in the present study was designed originally to test the idea that the shape selectivity of IT neurons reflects the computational challenges posed when differentiating objects at different orientations in depth [12,14]. Here, we show that deep CNNs that were trained to classify a large set of natural images show response modulations to these shapes that are similar to those observed in macaque IT neurons. We show that untrained CNNs with the same architecture than the trained CNNs, but with random weights, demonstrate a poorer IT-CNN similarity than the CNNs trained in classification. The difference between the trained and untrained CNNs emerged at the deep convolutional layers, where the similarity between the shape-related response modulations of IT neurons and the trained CNNs was high. Unlike macaque IT neurons, human similarity judgements of the same shapes correlated best with the deepest layers of the trained CNNs.

Early and many later studies of IT neurons employed shapes as stimuli (e.g. [26–31,22,32–37]), in keeping with shape being an essential object property for identification and categorization. Deep CNNs are trained with natural images of objects in cluttered scenes. If deep CNNs are useful models of biological object recognition [38], their shape representations should mimic those of the biological system, although the CNNs were not trained with such isolated shapes. We show here that indeed the representation of the response modulations by rather abstract, unnatural shapes is highly similar for deep CNN layers and macaque IT neurons. Note that the parameters of these CNN models are set via supervised machine learning methods to do a task (i.e. classify objects) rather than to replicate the properties of the neural responses, as done in classic computational modeling of neural selectivities. Thus, the same CNN model that fits neural responses to natural images [1–4] also simulates the selectivity of IT neurons for abstract shapes, demonstrating that these models show generalization across highly different stimulus families. Of course, the high similarity between deep CNN layers and IT neurons activation patterns we show here may not generalize for (perhaps less fundamental) shape properties that we did not vary in our study.

Kubilius et al. [39] showed that deep nets captured shape sensitivities of human observers. They showed that deep Nets, in particular their deeper layers, show a NAP advantage for objects (“geons”), as does human perception (and macaque IT [18]). Although we also manipulated NAPs, our shapes differed in addition in other properties such as regularity and complexity. Furthermore, our shapes are unlike real objects and more abstract than the shaded 3D objects employed by Kubilius et al. [39] when manipulating NAPs.

In both the representational similarity analysis and the response modulations comparisons amongst shape groups, we found that the correspondence between IT and deep CNN layers peaked at the deep convolutional layers and then decreased for the deeper layers. Recently, we observed a similar pattern when using deep CNN activations of individual layers to model the shape selectivity of single neurons of the middle Superior Temporal Sulcus body patch [4], a fMRI-defined region of IT that is located posterior with respect to the present recordings. The increase with deeper layers of the fit between CNN activations and neural responses has also been observed when predicting with CNN layers macaque IT multi-unit selectivity [40], voxel activations in human LO [9] and the representational similarity of macaque and human (putative) IT [8,10] using natural images. However, the decrease in correlation between CNNs and neural data that we observed for the deepest layers was not found in fMRI studies that examined human putative IT [8,10], although such a trend was present in [6] when predicting CNN features from fMRI activations. The deepest layers are close to or at the categorization stage of the CNN and hence strongly dependent on the classifications the network was trained on. The relatively poor performance of the last layers is in line with previous findings that IT neurons show little invariance across exemplars of the same semantic category [41,42], unlike the deepest CNN units [43].

The question of what the different layers in the various CNN models with different depths represent neurally remains basically unanswered. Shallow CNN layers can be related to early visual areas (e.g. V1; V4) and deeper layers to late areas (e.g. IT). However, different laminae within the same visual area (e.g. input and output layers) may also correspond to different layers of CNNs. Furthermore, units of a single CNN layer may not correspond to a single area, but the mapping might be more mixed with some units of different CNN layers being mapped to area 1, while other units of partially overlapping CNN layers to area 2, etc. Finally, different CNN layers may represent different temporal processing stages within an area, although this may map partially to the different laminae within an area. Further research in which recordings in different laminae of several areas will be obtained for the same stimulus sets, followed by mapping these to units of different layers in various CNNs, may start to answer this complex issue.

In contrast with IT neurons, human similarity judgements of our shapes matched to a greater extent the last rather than the less deep convolutional layers. In particular, the deepest layers showed a similar enhanced sensitivity for straight versus curved irregular shapes. The untrained CNNs did not show such straight versus irregular bias for the irregular shapes. Thus, it appears that a system, be it artificial like the CNNs or a biological system like humans, that is required to classify natural images of objects develops such bias for curved versus straight contours, indicating that this shape property must be highly informative for object categorization. Whether this relates to straight versus curved being a NAP [14] is unclear.

Kayaert et al. [12] employed a sorting task to rate shape similarity. In this task, subjects were required to sort the shapes into groups based on their similarity. Although this is not the same as labeling an object, the task for which the CNNs were trained, higher order classification can intrude the sorting task judgements. This may explain why the human sortings of Kayaert et al. [12] resembled that closely the activation pattern seen at the deepest CNN layers, which are strongly category label driven. Interestingly, even for the shallow convolutional layers, the correlations between the human judgements and the CNN activations were higher for the trained compared with the untrained CNNs. This contrasted with the equal correlations for trained and untrained shallow layers for the IT data. This suggests that the trained shallow CNN layers show already some, albeit weak, bias for higher order category-related information.

Previous studies that compared deep CNNs and neural responses rarely included untrained CNNs as control (e.g. [8], [40]). We found the untrained CNNs helpful in interpreting our data. The comparison with untrained CNNs can inform to what extent neural responses reflect features that can be picked up by untrained CNNs (because of CNN architectural properties such as tiling of local RFs in shallow layers and non-linear pooling). Indeed, we found that most layers of the untrained CNNs represented rather closely the pixel-based graylevel differences between the shape groups, which assisted to interpret the representational similarity of the trained CNNs at shallow layers. Thus, we advise that future studies use untrained CNNs as control or benchmark.

Currently, deep CNNs are the best models we have of primate object recognition, providing the best quantitative fits of ventral stream stimulus selectivities and primate recognition behavior [38]. However, recent studies show that CNNs have their limitations, especially when stimuli are noisy or partially occluded. For instance, the commonly used deep CNNs tolerate less image degradation than humans [44], can be fooled by unrecognizable images [45] or show a sensitivity to imperceptible stimulus perturbations (“adversarial examples”; [46]). Our data show that training CNNs in object categorization produces at least some shape selectivities (that are thought to reflect fundamental aspects of shape processing [14]) similar to those that are observed in neural IT data and human similarity judgements. This does not imply that CNNs can explain all shape or stimulus selectivity in IT and there is still considerable room for model improvement (e.g. recurrent connectivity etc.).

In conclusion, deep CNN layers that were trained to classify complex natural images represented differences among relatively simple abstract 2D-shapes similar to macaque IT neurons. Human sorting of the same shapes corresponded better with the deepest layers of the CNNs. The similarity between IT neurons and the deeper convolutional layers is greater for trained compared to untrained CNNs, suggesting a role of image classification in shaping the shape selectivity of macaque IT neurons. The latter likely occurs during ontogenetic development, but may not result from the same supervised learning algorithm as employed to train the CNNs. Indeed, independent of the particular training protocol (e.g. supervised versus unsupervised), any biological object classification system may have similar shape representation biases that are inherently useful for performing invariant object classification.

## Materials and methods

### Ethics statement

Two male rhesus monkeys served as subjects. The animals were housed individually with visual and auditory contact with conspecifics. During the recording weeks, they had controlled access to fluids but food was available at libitum. All procedures were in accordance with the Weatherall report on “The use of non-human primates in research” and were approved by the Animal Ethics committee of the KU Leuven (protocol number: P631/2002).

### Stimuli

The 64 shapes were identical to the first stimulus set employed by Kayaert et al. [12] and are shown in Fig 1. The Regular shapes R were created with Studio MAX, release 2.5, while the Irregular shapes were made with Fourier Boundary Descriptors, using MATLAB, release 5. The Irregular Simple Straight (ISS) stimuli were made by replacing the curves of the Irregular Simple Curved (ISC) shapes by straight lines while preserving the overall shape. The increase in complexity of the Irregular Complex (IC) shapes compared to the simpler ISC shapes was produced by increasing the number and frequency of the Fourier Boundary Descriptors.

Each group contains 8 pairs of stimuli (one stimulus in row a and one in row b in Fig 1). The columns of Fig 1 comprise a set of 4 pairs (one for each group) that were matched in overall size and aspect ratio, both within and between groups. The averaged pixel-based graylevel differences between the members of the pairs were balanced across groups (see [12] for more details). The members of the pairs within the Regular shapes differ in a NAP, such as parallel vs. nonparallel sides, or straight vs. curved contours. The differences among the members of an irregular pair were created by varying the phase, frequency or amplitude of the Fourier Boundary Descriptors. For the single unit recordings and the human behavioral study, all stimuli were filled with the same random dot texture pattern. The number of black and white dots was required to be equal for 2*2 squares of pixels, so the texture patterns were highly uniform. Stimuli were presented on a gray background. In the single unit study, they extended approximately 7 degrees and were shown at the center of the screen. We employed the identical shapes for the CNN modeling, except that the noise pattern was replaced by a uniform white surface (see Fig 1 for the actual stimuli presented to the CNNs).

### Electrophysiology data of monkey IT

The single unit data have been published before [12] and the procedures have been described in detail in that paper. Therefore, we will summarize here only briefly the experimental procedures. The IT recordings were made while the two monkeys performed a passive fixation task. Eye movements were measured with the scleral search coil technique or with a noninvasive eye tracker (ISCAN). During the recordings, their head was fixed by means of an implanted head post. We employed the standard dorsal approach to IT and recording sites were verified with MRI and CT scans with the guiding tube in situ. We lowered a tungsten microelectrode through the guiding tube that was fixed in a Crist grid, which was positioned within the plastic recording chamber. The signals of the electrode were amplified and filtered using standard single-cell recording equipment. Single units were isolated on line and their timing was stored together with stimulus and behavioral events for later offline analysis.

The stimuli were presented during fixation for 200 ms in a randomly interleaved fashion. In the present study, the response of a neuron was defined as the average firing rate in spikes/s during a time interval of 250 ms, starting from 50 to 150 ms after stimulus onset. The starting point of this time interval was chosen for each neuron to best capture its response, by inspection of the peristimulus time histograms averaged across the stimuli. Responses were averaged across presentations per stimulus. The minimum number of presentations per stimulus was 5 (median = 10). The data set consisted of 119 anterior IT neurons (76 in monkey 1 and 43 in monkey 2) that showed significant response selectivity to the stimuli of the set (ANOVA, p<0.05). The data were pooled across animals. The neurons were located in the lower bank of the Superior Temporal Sulcus and the lateral convexity of anterior IT (TEad).

### Sorting task in human subjects

As described by Kayaert et al. [12], printed versions of the shapes were given to 23 naive adult human subjects who were asked to sort the stimuli in groups based on shape similarity. No further definition of similarity was given and they could freely choose the number of groups. This is a classical task to measure image similarities [47]. Dissimilarity values between pairs of stimuli were computed by counting the number of subjects that put the two members in different groups.

### Deep convolutional neural networks

In order to compare the shape representation of the IT neurons’ population with deep CNN layers, we extracted stimulus features for each processing stage (layer) of three deep models: Alexnet [20], VGG-16 and VGG-19 [21]. We used the pretrained networks, which are available through the MatConvNet toolbox [48] in MATLAB, and their untrained versions. The pretrained CNNs were trained on ~1.2 million natural images divided in 1,000 classes for the ImageNet Large Scale Visual Recognition Challenge 2012 (ILSVRC2012). The untrained versions of these networks have the same architecture, but did not undergo any training, thus no update of their weights took place after initialization. Their layer weights were initialized by sampling randomly from a normal distribution, using the opts.weightInitMethod = 'gaussian' setting in the cnn_imagenet.m function of the MatConvNet toolbox. The stimuli shown to the CNNs were black and white images with pixel values ranging from 0–255 (0 for black and 255 for white). Before feature extraction, the mean of the ILSVRC2012 training images was subtracted from each stimulus, since this was also part of the preprocessing stage of the networks’ training procedure. In addition, the stimuli were rescaled accordingly to match each network’s input requirements (227x227 pixels for Alexnet and 224x224 pixels for VGG-16 & VGG-19).

#### Alexnet

This deep CNN by Krizhevsky et al. [20] was the first successful deep CNN that outperformed existing object recognition algorithms and won first place at the ILSVRC2012. It incorporates multiple processing stages (or layers) in 8 weight layer groups, including convolutional, Rectified Linear Units (RELU), normalization, max pooling and fully connected layers. The MatConvNet pretrained version of Alexnet on the validation data of ILSVRC2012 had a 42.6% top-1 error rate performance and 19.6% top-5 error rate.

#### VGG-16 & VGG-19

Introduced in 2014 [21], they are deeper CNNs than Alexnet and consist of 16 and 19 weight layers, respectively, including convolutional, RELU, max pooling and fully connected layers. The pretrained versions of MatConvNet follow the same principles as Alexnet in terms of their training procedure and processing stages, but they don’t include a normalization layer due to lack of performance improvement on the ILSVRC dataset and increased computation time and memory consumption [21]. The MatConvNet versions of VGG-16 and VGG-19 had 28.7% and 28.5% top-1 error rate performance, respectively, and a 9.9% top-5 error rate performance for both on the validation data of ILSVRC2012.

### Data analyses

In all analyses, we employed as distance metric the normalized Euclidean distance between the neuronal responses or deep CNN unit activations:

$${\left(\frac{\Sigma_{i}^{n}{\left(R_{i}^{1}-R_{i}^{2}\right)}^{2}}{n}\right)}^{\frac{1}{2}},$$

where $R_{i}^{1}$ is the response of neuron or deep CNN unit *i*, to stimulus 1, and *n* is the number of neurons or the number of deep CNN units in a specific layer. For the representational similarity analyses, we computed also a second distance metric: 1- Spearman’s correlation coefficient. The Spearman rank correlation coefficient ρ was computed between the neural responses or CNN units’ activations for all stimulus pairs.

To compare neuronal data to CNN layers, we performed representational similarity analysis [49], using both distance metrics. We constructed representational dissimilarity matrices (RDMs) for the whole stimulus set (n = 64 stimuli) for both IT neurons and each deep CNN layer (trained and untrained separately; for examples see Fig 3), by arranging all possible pairwise distances in 64x64 RDMs. We extracted all values above the diagonal (upper triangle of the RDM, excluding the diagonal) of the symmetrical RDMs, and computed for each layer the Spearman rank correlation coefficient between the distances of the corresponding pairs of the neural and CNN matrices.

We computed 95% confidence intervals of the Spearman correlation coefficient between neural and CNN distances by resampling with replacement 10,000 times 119 neurons out of our pool of IT neurons and correlating each time the resulting neural distance matrix with each deep CNN layer for the trained and untrained versions of the same network. The confidence intervals corresponded to the 2.5 and 97.5 percentiles of the bootstrapped correlation coefficient distributions. To assess whether the trained deep CNN layers significantly differed from the untrained, we computed for each CNN layer the distribution of the paired differences of trained minus untrained layer correlations across the 10,000 iterations (one difference per bootstrapped neuronal sample). For each layer, we computed the percentile in the corresponding distribution of the zero difference value and these defined the p values of the test. For each of the 3 CNNs, we corrected the p values for multiple comparisons (n = number of CNN layers) using the Benjamini and Hochberg [50] False Discovery Rate (FDR) procedure. A difference between the trained and untrained CNNs was judged to be significant when FDR q < 0.05. The same procedure was used to assess the significance of the difference in IT-CNN correlations between the original and reduced shape size for each of the CNN layers (Fig 5). We employed a similar procedure to test the significance of the difference in Spearman rank correlation coefficients of the neural and CNN distances between the first layer and each subsequent layer. Thus, we computed the pairwise difference between the correlation for the first and a subsequent layer for each of the 10,000 bootstrapped neural samples and then obtained the percentile of the zero difference in that distribution of differences. The p values were corrected for multiple comparisons using the FDR procedure and significance was defined when q < 0.05.

In the second analysis, using only the original, non-bootstrapped distances, we compared the pairwise Euclidean stimulus distances amongst the 4 stimulus groups R, IC, ISC, ISS. For each group, we included only the stimulus pairs numbered 1–8 in Fig 1, i.e. for each group the members of the a and b rows of Fig 1. In addition, we selected the distances for the “ISCa vs. ISSa” and “ISCb vs. ISSb” pairs of Fig 1, e.g. the column-wise distances between row a of ISC and row a of ISS in Fig 1 (likewise for the b rows). This produced twice 8 distances for the ISC versus ISS comparison, which we analyzed separately, unlike in Kayaert et al. [12]. For each of the 4 groups and the two ISC versus ISS comparisons, we computed the mean distance (and standard errors of the mean) across the 8 pairs per group or comparison. To quantify the relationship between the mean distances across groups for the neural data and each CNN layer, we computed the Pearson correlation coefficient between the mean neural distances and the mean distances of the CNN layers (n = 6 pairs of distances per layer). A similar analysis was performed comparing the CNN layer distances and the distances based on the human ratings. However, for this analysis, the available human rating data consisted of the distances that were computed by Kayaert et al [12], having an ISC versus ISS comparison of 8 stimulus pairs (for selection of those pairs, see [12]) instead of twice 8 pairs as above. We compared those distances with the average of the “ISCa vs. ISSa” and “ISCb vs. ISSb” pairs of the CNN layers. Note that our average neural distances for the “ISCa vs. ISSa” and “ISCb vs. ISSb” pairs were highly similar to those for the 8 “ISC vs. ISS” pairs selected by Kayaert et al. [12], justifying this procedure.

We compared neural dissimilarities also with dissimilarities based on pixel graylevels and the HMAX model [5], employing the same procedures as in Kayaert et al.. We computed the Euclidean distance between the gray-level values of the pixels for all image pairs (Fig 3B). In addition, we computed the Euclidean distances between the outputs of C2-units of the HMAX model as described by Riesenhuber and Poggio [5] and presented in [12]. The HMAX C2 units were designed to extract moderately complex features from objects, irrespective of size, position and their relative geometry in the image. HMAX-based dissimilarities were computed as the Euclidean distance between the output of the 256 C2 units.

## Supporting information

S1 Fig Representational similarity analysis of deep CNN layers and IT neurons for the whole shape set. Spearman rank correlation coefficients between IT and model layer similarities are shown for each layer of the three CNN models used. The same analysis was performed for two distance metrics: Euclidean distance (left column) and 1-Spearman rank correlation (right column). Error bars depict 95% confidence intervals, determined by 10,000 bootstrap samples of the IT neuron pool (n = 119 neurons). Stars indicate layers for which the Spearman rank correlations for the trained version differed significantly from its untrained version (paired bootstrap test (see Materials and Methods); False Discovery Rate corrected q<0.05). Crosses indicate trained layers which differed significantly from the first convolutional layer of the network (paired bootstrap test (see Materials and Methods); False Discovery Rate corrected q<0.05). Layers are indicated by the same nomenclature as in Fig 2 of the main text. The horizontal line and gray band indicate the median and 95% interval, respectively, of the Spearman-Brown corrected split-half correlations (n = 10000 splits) of the neuronal distances, as an estimate of the noise ceiling.
(TIF)

S2 Fig Representational similarity analysis of deep CNN layers and IT neurons for the whole shape set with two different sizes. Spearman rank correlation coefficients between IT and model layer similarities are shown for each layer of the three CNN models for the original and twofold smaller sizes (“reduced size”). The dissimilarities were Euclidean distances. Error bars depict 95% confidence intervals, determined by 10,000 bootstrap samples of the IT neuron pool (n = 119 neurons). Stars indicate layers for which the Spearman rank correlations for the trained version differed significantly from its untrained version (paired bootstrap test; False Discovery Rate corrected q<0.05). The horizontal line and gray band indicate the median and 95% interval, respectively, of the Spearman-Brown corrected split-half correlations (n = 10000 splits) of the neuronal distances, as an estimate of the noise ceiling.
(TIF)

S3 Fig Similarities between IT and CCN peak layer shape dissimilarities as a function of percent of units. (A) Spearman rank correlation coefficients between IT and peak CNN layer similarities are shown for each of the three CNN models as a function of sample size, expressed as percentage of the total number of units that were activated differentially by the 64 shapes. (B) Pearson correlation coefficients between the mean neural distances and the mean distances of the peak CNN layer (n = 6 mean distances; Fig 8 of the main text) as a function of percentage of the total number of units. The total number of units (100%) for each CNN layer is listed in the legend. Note that 0.1% corresponds to only 3 Alexnet units, explaining the large range of correlations for that sample size. The dissimilarities were Euclidean distances. Error bars depict 95% confidence intervals, determined by 10,000 random samples from the population of differentially activated CNN units of that layer.
(TIF)

S4 Fig Similarities between IT and CNN peak layer shape dissimilarities as a function of retained Principal Components. Top left panels: Spearman rank correlation coefficients between IT and peak CNN layer similarities are shown for each of the three CNN models as a function of retained principal components of the CNN layer activations. The dissimilarities were Euclidean distances. Error bands depict 95% confidence intervals, determined by 10,000 bootstrap samples of the IT neuronal pool. Top right panel: The cumulative proportion of explained variance as a function of principal component number for the 3 CNN. Bottom panels: Pearson correlation coefficients between the mean neural distances and the mean distances of the peak CNN layer (n = 6 mean distances; see Fig 10 of the main text) as a function of retained principal components. The error bands represent 95% confidence intervals, determined by 10,000 bootstrap samples of the IT neuronal pool.
(TIF)

S5 Fig Correspondence between model dissimilarities and biological dissimilarities (IT responses and human judgement-based dissimilarities) for the shape groups. (A, B, C). Gray curves show the Pearson correlation coefficients between the mean neural distances and the mean distances of the CNN layers (n = 6 mean distances per layer). Blue curves show the Pearson correlation coefficients of the CNN layer distances and the distances based on human judgements. Data for trained and untrained CNNs are plotted with full and dashed lines, respectively. Nomenclature of CNN layers as in Fig 2 of the main text. Results for all three models (Alexnet, VGG-16 and VGG-19) are displayed in the subplots (A, B and C). (D) Neural: Pearson correlation coefficient between the mean IT distances and the mean distances of the peak Alexnet layer, peak VGG-16 layer, VGG-19 layer, the mean HMAX C2 layer distances, and mean pixel-based distances, across shape groups. Human: Pearson correlation coefficient between the distances based on the human judgements and the peak Alexnet layer, peak VGG-16 layer, peak VGG-19 layer, HMAX C2 layer and pixel-based distances.
(TIF)
