# Supplementary figures and images for: Representations of regular and irregular shapes by deep Convolutional Neural Networks, monkey inferotemporal neurons and human judgments
Source: PLoS Comput Biol. 2018 Oct 26;14(10):e1006557. doi: 10.1371/journal.pcbi.1006557 (PMC6231692; doi:10.1371/journal.pcbi.1006557)

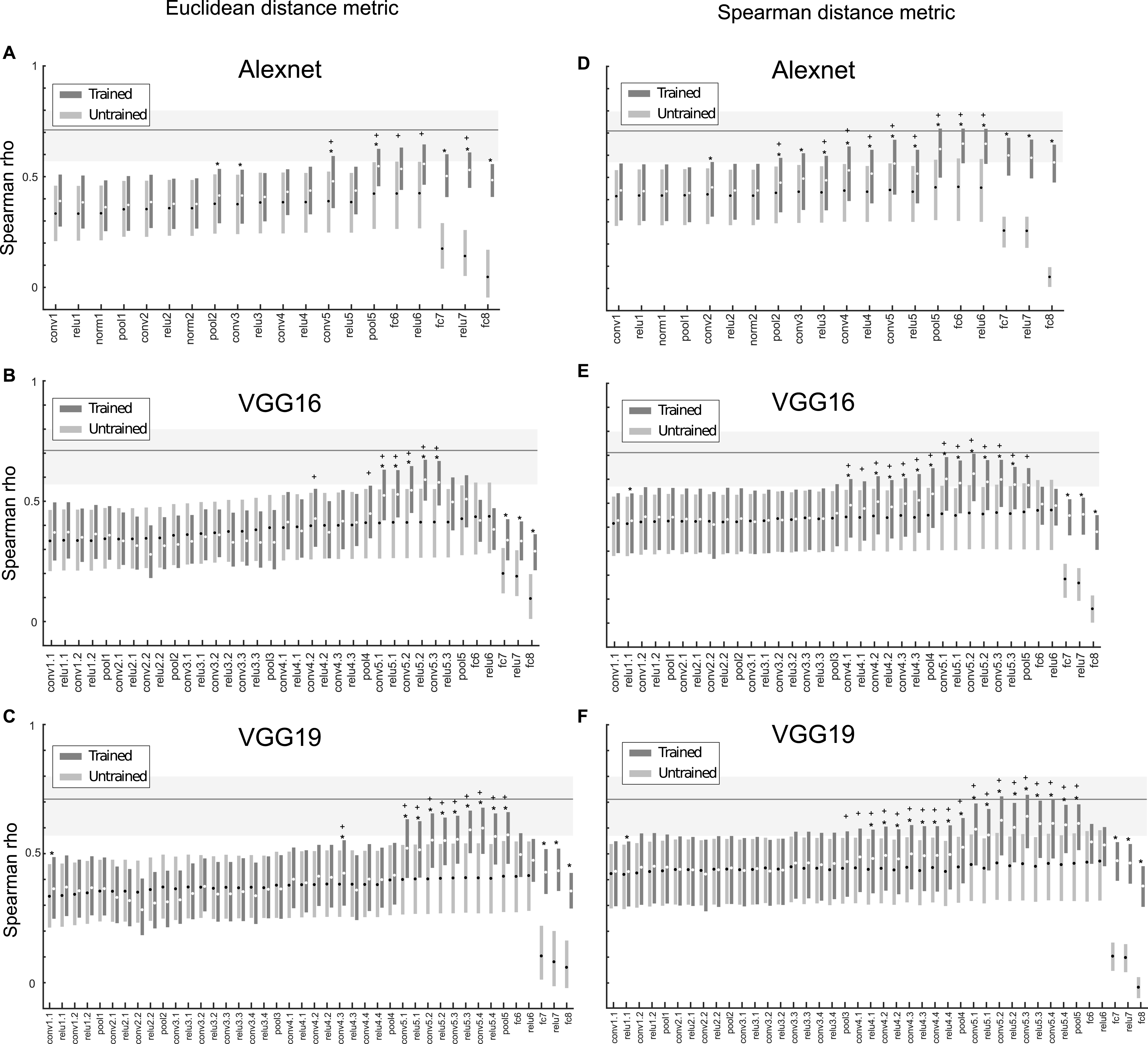

Supplement: S1 Fig — Spearman rank correlation coefficients between IT and model layer similarities are shown for each layer of the three CNN models used. The same analysis was performed for two distance metrics: Euclidean distance (left column) and 1-Spearman rank correlation (right column). Error bars depict 95% confidence intervals, determined by 10,000 bootstrap samples of the IT neuron pool (n = 119 neurons). Stars indicate layers for which the Spearman rank correlations for the trained version differed significantly from its untrained version (paired bootstrap test (see Materials and Methods); False Discovery Rate corrected q<0.05). Crosses indicate trained layers which differed significantly from the first convolutional layer of the network (paired bootstrap test (see Materials and Methods); False Discovery Rate corrected q<0.05). Layers are indicated by the same nomenclature as in Fig 2 of the main text. The horizontal line and gray band indicate the median and 95% interval, respectively, of the Spearman-Brown corrected split-half correlations (n = 10000 splits) of the neuronal distances, as an estimate of the noise ceiling. (TIF) [file pcbi.1006557.s001.tif]

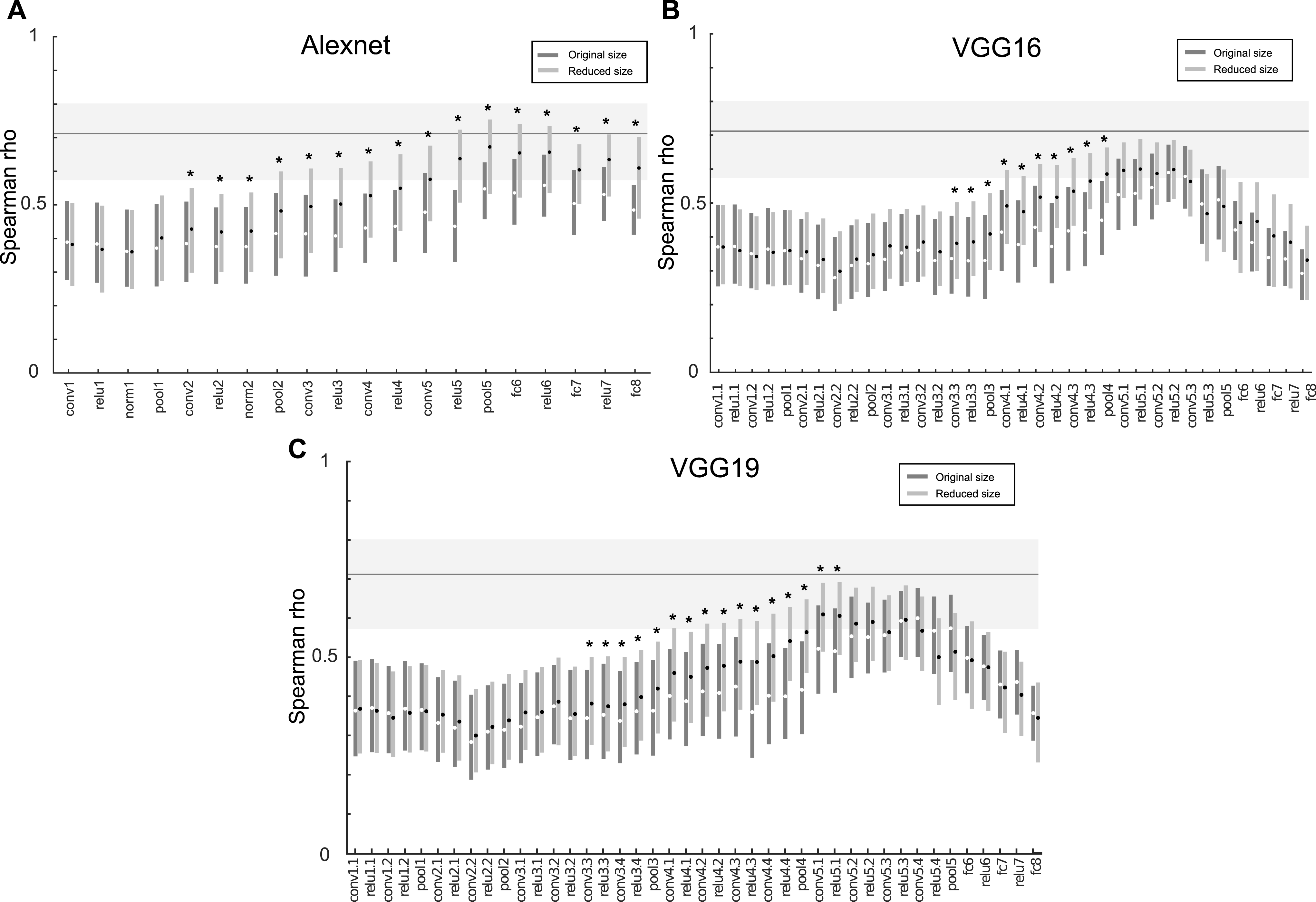

Supplement: S2 Fig — Spearman rank correlation coefficients between IT and model layer similarities are shown for each layer of the three CNN models for the original and twofold smaller sizes (“reduced size”). The dissimilarities were Euclidean distances. Error bars depict 95% confidence intervals, determined by 10,000 bootstrap samples of the IT neuron pool (n = 119 neurons). Stars indicate layers for which the Spearman rank correlations for the trained version differed significantly from its untrained version (paired bootstrap test; False Discovery Rate corrected q<0.05). The horizontal line and gray band indicate the median and 95% interval, respectively, of the Spearman-Brown corrected split-half correlations (n = 10000 splits) of the neuronal distances, as an estimate of the noise ceiling. (TIF) [file pcbi.1006557.s002.tif]

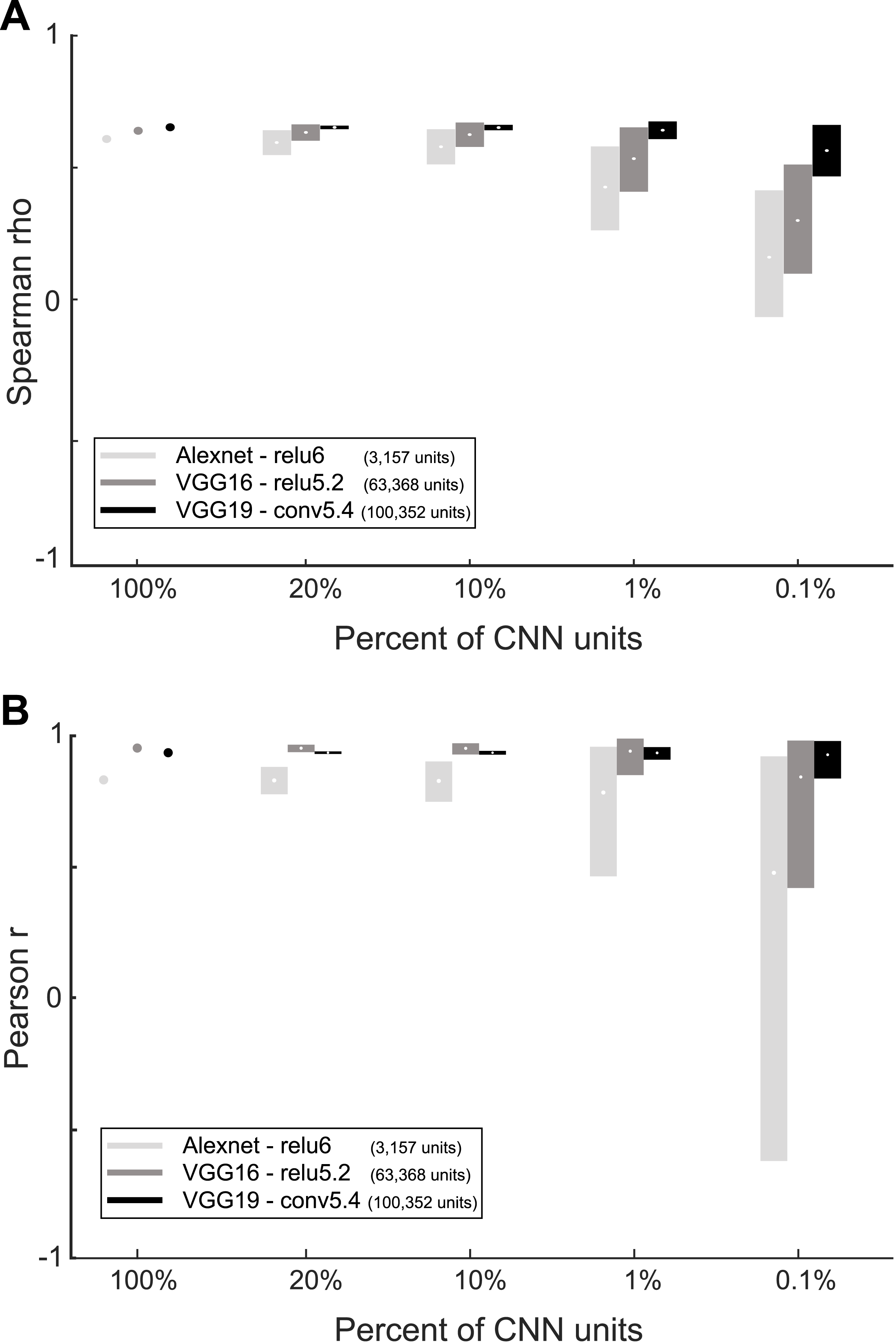

Supplement: S3 Fig — (A) Spearman rank correlation coefficients between IT and peak CNN layer similarities are shown for each of the three CNN models as a function of sample size, expressed as percentage of the total number of units that were activated differentially by the 64 shapes. (B) Pearson correlation coefficients between the mean neural distances and the mean distances of the peak CNN layer (n = 6 mean distances; Fig 8 of the main text) as a function of percentage of the total number of units. The total number of units (100%) for each CNN layer is listed in the legend. Note that 0.1% corresponds to only 3 Alexnet units, explaining the large range of correlations for that sample size. The dissimilarities were Euclidean distances. Error bars depict 95% confidence intervals, determined by 10,000 random samples from the population of differentially activated CNN units of that layer. (TIF) [file pcbi.1006557.s003.tif]

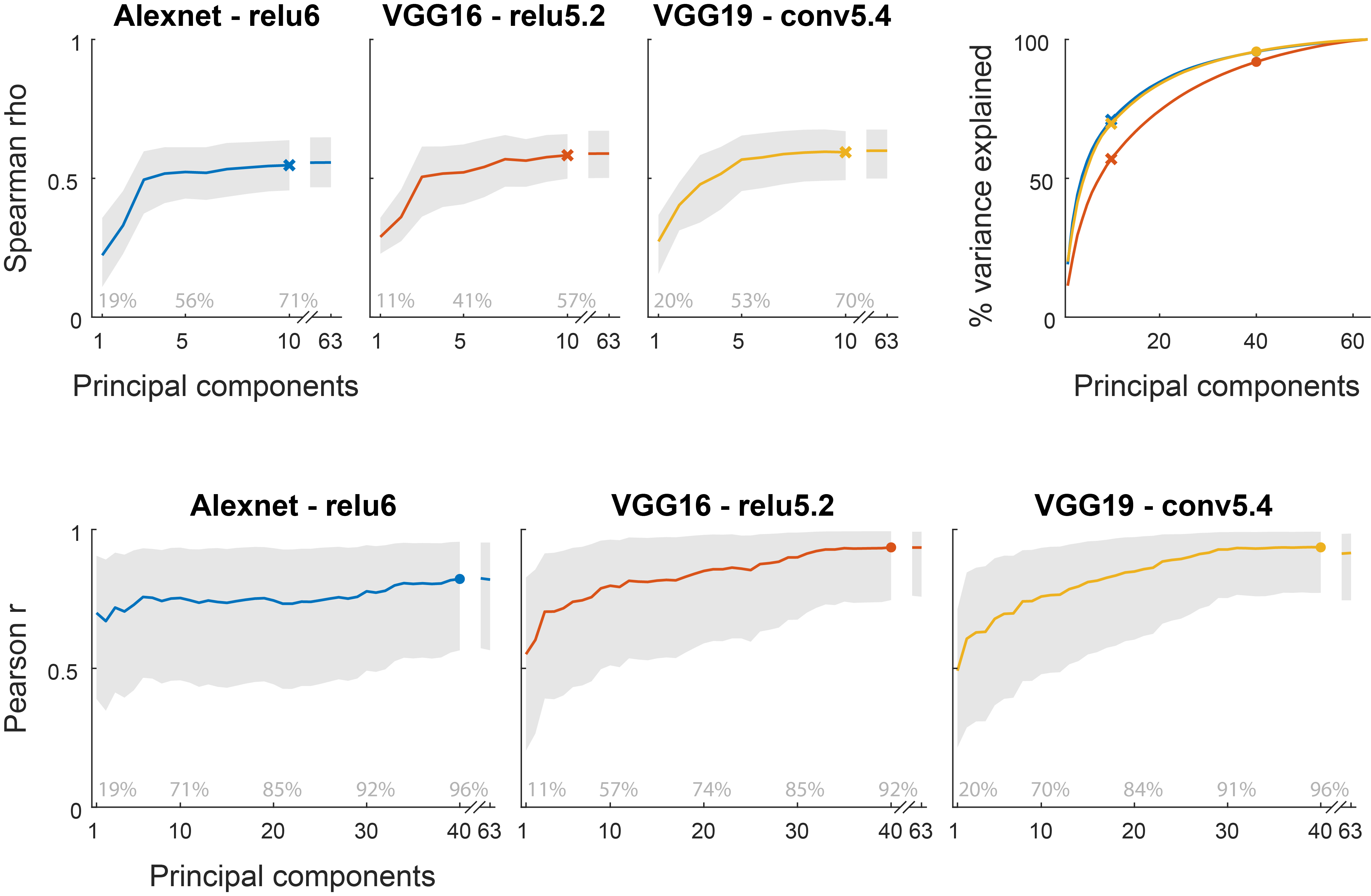

Supplement: S4 Fig — Top left panels: Spearman rank correlation coefficients between IT and peak CNN layer similarities are shown for each of the three CNN models as a function of retained principal components of the CNN layer activations. The dissimilarities were Euclidean distances. Error bands depict 95% confidence intervals, determined by 10,000 bootstrap samples of the IT neuronal pool. Top right panel: The cumulative proportion of explained variance as a function of principal component number for the 3 CNN. Bottom panels: Pearson correlation coefficients between the mean neural distances and the mean distances of the peak CNN layer (n = 6 mean distances; see Fig 10 of the main text) as a function of retained principal components. The error bands represent 95% confidence intervals, determined by 10,000 bootstrap samples of the IT neuronal pool. (TIF) [file pcbi.1006557.s004.tif]

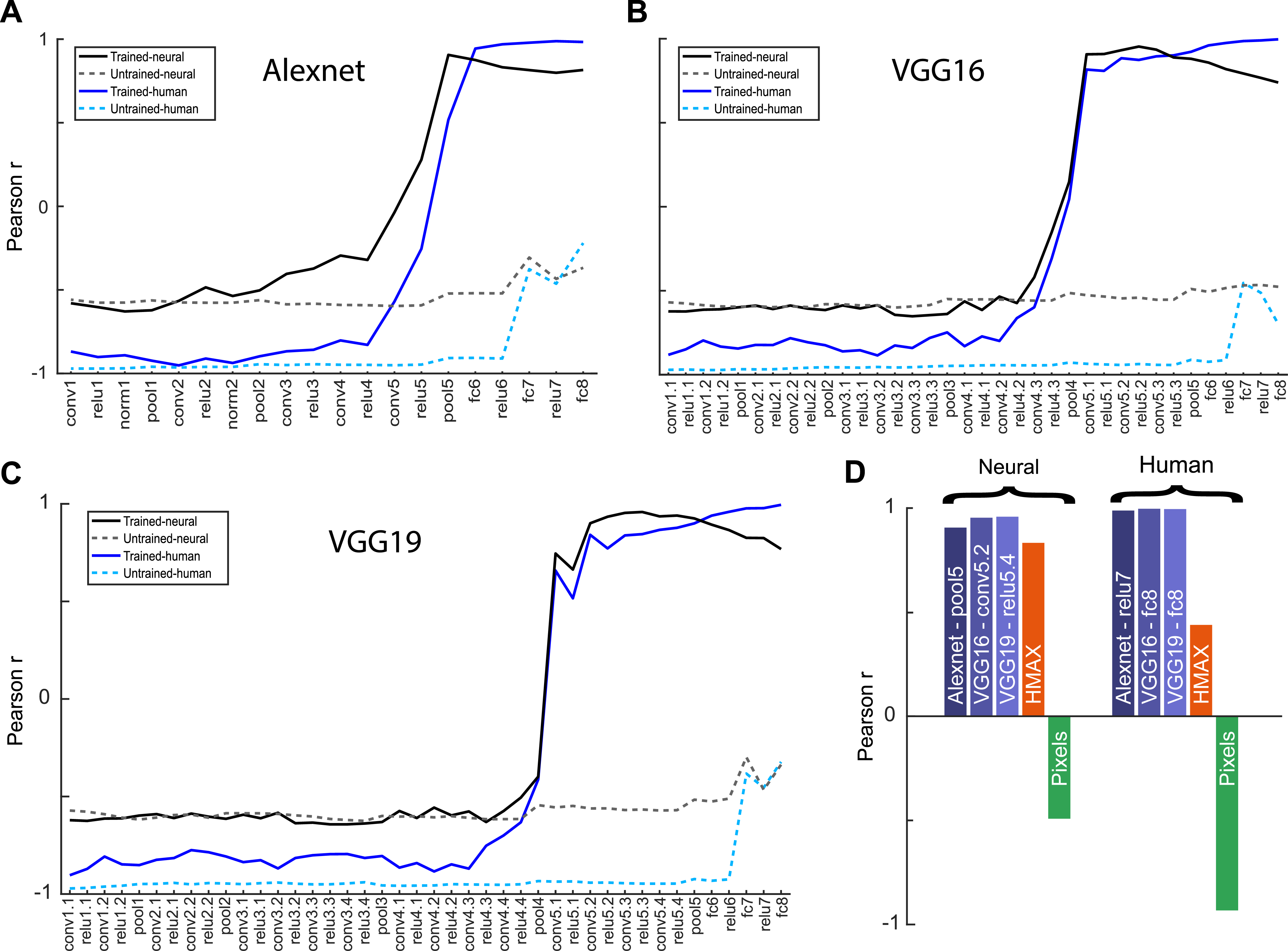

Supplement: S5 Fig — (A, B, C). Gray curves show the Pearson correlation coefficients between the mean neural distances and the mean distances of the CNN layers (n = 6 mean distances per layer). Blue curves show the Pearson correlation coefficients of the CNN layer distances and the distances based on human judgements. Data for trained and untrained CNNs are plotted with full and dashed lines, respectively. Nomenclature of CNN layers as in Fig 2 of the main text. Results for all three models (Alexnet, VGG-16 and VGG-19) are displayed in the subplots (A, B and C). (D) Neural: Pearson correlation coefficient between the mean IT distances and the mean distances of the peak Alexnet layer, peak VGG-16 layer, VGG-19 layer, the mean HMAX C2 layer distances, and mean pixel-based distances, across shape groups. Human: Pearson correlation coefficient between the distances based on the human judgements and the peak Alexnet layer, peak VGG-16 layer, peak VGG-19 layer, HMAX C2 layer and pixel-based distances. (TIF) [file pcbi.1006557.s005.tif]
